# Application of an updated methodology to estimate the burden of healthcare-associated infections in Italy, 2022

**DOI:** 10.2807/1560-7917.ES.2025.30.18.2400812

**Published:** 2025-05-08

**Authors:** Costanza Vicentini, Valerio Bordino, Luca Bresciano, Stefania Di Giacomo, Fortunato D’Ancona, Carla Maria Zotti, Italian PPS network, Italian PPS network, Antonella Agodi, Italo Francesco Angelillo, Federico Argiolas, Luca Arnoldo, Leonardo Ascatigno, Silvia Atti, Pamela Barbadoro, Martina Barchitta, Stefania Bellio, Fabrizio Bert, Aida Bianco, Valentina Blengini, Margherita Boschetto, Roberta Bussolino, Alberto Carli, Giancarla Carraro, Luana Casanova, Marta Castagnotto, Paolo Castiglia, Adriana Cataldo, Danilo Cereda, Alessandro Cerrone, Roberto Cocconi, Lucia Crottogini, Paola Deambrosis, Giovanna Deiana, Manuela Di Giacomo, Heba Safwat Mhmoued Abdo Elhadidy, Luca Fabbri, Ugo Fedeli, Silvia Forni, Francesca Fortunato, Claudia Gastaldo, Irene Gintoli, Federico Grammatico, Pina Iannelli, Stefano Kusstatscher, Giulia Libero, Rosanna Loss, Susi Malerbi, Elisabetta Mantengoli, Domenico Martinelli, Horand Meier, Giuseppina Occhipinti, Dalia Palmieri, Paola Pau, Elisa Perri, Sergio Pili, Rosi Prato, Vincenzo Puro, Enrico Ricchizzi, Giancarlo Ripabelli, Edoardo Rolfini, Antonino Russotto, Mario Saia, Camilla Sticchi, Pierina Rita Tanchis, Elena Vecchi

**Affiliations:** 1 Department of Public Health and Paediatrics, University of Turin, Italy; 2 Prevention Department, Local Health Trust TO5, Turin, Italy; 3 Epidemiology, Biostatistics and Mathematical Modelling Unit (EPI), Department of Infectious Diseases, Istituto Superiore di Sanità (ISS), Rome, Italy; 4 The members of the network are listed under Collaborators

**Keywords:** Point prevalence survey, healthcare associated infections, burden, DALYs, Italy

## Abstract

**Background:**

Accurate burden estimates are necessary to inform priority setting and rational resource allocation. Weighting prevalence inversely proportional to time-at-risk has been proposed as a solution for length-biased sampling, an important limitation affecting prevalence to incidence conversion for healthcare-associated infections (HAIs).

**Aim:**

This study aimed to update Italian burden estimates by calculating HAI incidence, attributable mortality and disability-adjusted life years (DALYs). Further, we describe an adapted methodology for burden estimations.

**Methods:**

We used data from the latest European Centre for Disease Prevention and Control (ECDC) point prevalence survey (PPS) of HAIs, conducted in Italy in November 2022, to calculate the burden of five major HAIs at national level. We adapted the Burden of Communicable Diseases in Europe (BCoDE) methodology to include inverse probability weighting and compared results of naïve and weighted calculations.

**Results:**

The national sample included 18,397 patients. Overall, 564.8 DALYs per 100,000 general population resulted from weighted calculations (95% uncertainty interval (UI): 450.04–694.38), with an annual incidence of 685.42 cases per 100,000 general population (95% UI: 611.09–760.86) and 33.23 deaths per 100,000 general population per year (95% UI: 28.62–38.33). Concerning naïve estimates, overall 1,017.81 DALYs per 100,000 general population were calculated (95% UI: 855.16–1,190.59). In both calculations, healthcare-acquired bloodstream infections had the highest burden in terms of DALYs per 100,000 hospitalised and general population.

**Conclusion:**

Our study confirmed the substantial burden of HAIs in Italy and renews the need to prioritise resources for infection prevention and control interventions.

**Key public health message**

**What did you want to address in this study and why?**

In Italy, healthcare-associated infections (HAIs) and infections caused by antimicrobial-resistant bacteria are considered a critical issue as well as a national priority due to their high rates. Accurately estimating their impact, in terms of frequency, number of deaths and disability, is essential to inform priority setting and rational allocation of healthcare resources.

**What have we learnt from this study?**

We developed and described in detail an updated, reproducible and fully transparent methodology for calculating HAI burden, which could lead to more accurate results.

**What are the implications of your findings for public health?**

This study confirmed the substantial burden of HAIs in Italy, in particular compared with other infectious diseases, and renews the need to prioritise resources for HAI prevention and control interventions.

## Introduction

The clinical and economic impact of healthcare associated infections (HAIs) on health systems is well documented [1,2]. In Italy, HAIs and infections caused by antimicrobial-resistant (AMR) pathogens in particular, are considered a critical issue as well as a national priority. Accurate burden estimates are necessary to inform priority setting and rational resource allocation. The European Centre for Disease Prevention and Control (ECDC) developed a methodology to estimate the burden of communicable diseases, including HAIs, in terms of disability-adjusted life years (DALYs), a composite metric which takes into account disease incidence, associated disabilities and years of life lost [1,3,4]. The Burden of Communicable Diseases in Europe (BCoDE) methodology proposed by ECDC is incidence-based, however, estimates for HAI burden have been obtained from prevalence data collected through the ECDC point prevalence survey (PPS) of HAIs in acute-care hospitals [1,5].

Based on Italian PPS data in 2017, we previously estimated a disease burden of more than 700 DALYs per 100,000 general population attributable to five HAIs [6]. We had hypothesised that the relatively higher burden obtained in our country in comparison with European estimates could be due to demographic characteristics of the Italian hospitalised population, to AMR levels, to the level of implementation of preventive strategies, or to other factors [1,6].

Compared with longitudinal surveillance, which allows to directly measure incidence rates, PPS are logistically less challenging to conduct and require less time and resources [7]. However, PPS are affected by limitations, including length-biased sampling [8]. We did not account for length bias in our previous HAI burden calculations, which could have affected results [6].

Weighting prevalence inversely proportional to time at risk has been proposed as a solution for length-biased sampling [8]. By applying inverse probability weighting to prevalence estimates of HAIs in intensive care units (ICUs), we recently obtained incidence estimates that did not differ significantly from those directly measured through longitudinal surveillance [9].

The aims of this study were: (i) to update Italian burden estimates by calculating HAI incidence, attributable mortality and DALYs based on the most recent PPS edition, conducted in 2022, and (ii) to propose an adapted methodology for burden calculations which incorporates inverse probability weighting and provides estimates corrected for length-biased sampling.

## Methods

### Study design, participants, and data collection

The third PPS in Italy (PPS3) was conducted in November 2022. For this study, we performed analyses considering data of the Italian representative sample. The Italian representative sample consisted of 59 hospitals from 19 Italian regions, totalling 19,735 patients. To reflect the regionalised structure of the Italian national health system [10], the sample size recommended by ECDC (55 hospitals) was distributed among Italian Regions, based on each Region’s proportion of population, number of acute-care hospital bed-days and number of discharges from acute care facilities, with each Region represented by at least one facility. Regions enrolled hospitals using convenience sampling, however, when more than one hospital per Region was required, facilities of different size were to be included.

The methodology for data collection has previously been described in detail [11,12]. Collected data at patient-level included age, sex, date of hospital admission, and severity of underlying medical conditions, which were assessed through the McCabe score. We used the McCabe score to adjust for life expectancy in disease models, which involves classifying patients in three categories according to disease prognosis: rapidly fatal (expected survival < 1 year), ultimately fatal (expected survival 1–4 years) and non-fatal (regular life expectancy). For patients with active HAIs, additional information was collected, including HAI type and date of symptom onset.

### Data processing for burden estimations – ECDC BCoDE methodology

For comparability with previous estimations [1,6], we considered in this study the same five HAI categories included in European and Italian studies of HAI burden using the ECDC BCoDE methodology: healthcare-associated pneumonia (HAP), healthcare-associated urinary tract infection (HA-UTI), healthcare-associated primary bloodstream infection HA-BSI (excluding neonatal BSIs), surgical site infection (SSI) and healthcare-associated *Clostridioides difficile* infection (HA-CDI), defined according to ECDC PPS case definitions [11].

**Figure 1:**
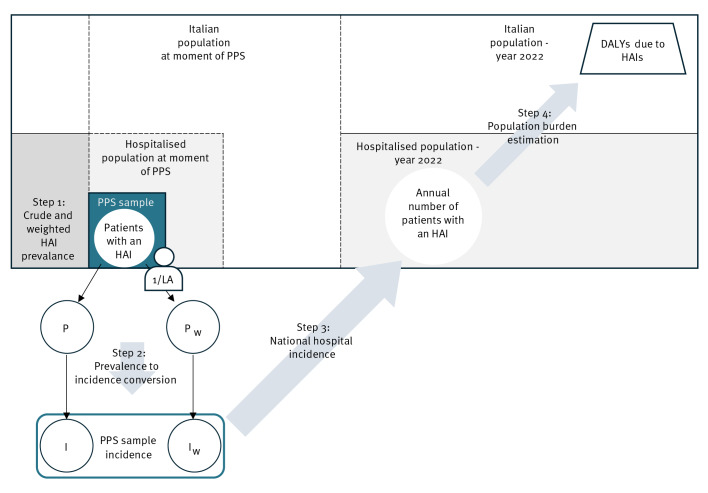
Overview of the workflow for healthcare-associated infections burden estimations, Italy, 2022

DALYs: disability-adjusted life years; I: incidence; I_w_: inverse probability-weighted incidence; LA: length of stay from admission to day of PPS; P: prevalence; P_w_: inverse probability-weighted prevalence; PPS: point prevalence survey.

As summarised in Figure 1, the workflow for obtaining burden estimations involved four steps, and was conducted using (i) crude and (ii) inverse probability-weighted prevalence estimates obtained from the Italian PPS3 representative sample. We calculated sex-, age- and McCabe stratum-specific incidence rates of the five considered HAIs from prevalence estimates using the Rhame and Sudderth Formula [13]. We obtained DALY estimates following the ECDC BCoDE methodology, with disease models adapted to the Italian population [1,3,4].

#### Step 1: Crude and weighted prevalence of healthcare-associated infections

We used data from the Italian PPS representative sample to estimate crude HAI prevalence (P) for each HAI type within each age, sex and McCabe score stratum, which was calculated as the number of patients with the considered HAI over the number of patients within each stratum.

We obtained weighted HAI prevalence (P_w_) by weighting each patient inversely proportional to their time at risk, i.e. length of hospital stay from admission to the day of the PPS (LA_i_ for patient i) [8]. Each patient in the representative sample was attributed a weight of 1/(LA_i_ + 1); weighted HAI prevalence for each HAI type was calculated as the sum of 1/(LA_i_ + 1) for patients with the considered HAI over the sum of 1/(LA_i_ + 1) for all patients within each stratum. For 14 patients (0.07% of the sample), LA_i_ could not be calculated due to missing or incorrect data, therefore these patients were excluded from the weighted analysis. We calculated mid-P Clopper–Pearson confidence intervals (CIs) for both P and P_w_.

#### Step 2: Naïve and inverse probability-weighted prevalence to incidence conversion – point prevalence survey sample

For each HAI type, we converted P and P_w_ into incidence per 100 admissions (I and I_w_ respectively) using an adapted version of the Rhame and Sudderth formula [13]:


$$I=P\;\times \frac{LA}{LN-INT}$$

$$I_{w}=P_{w}\;\times \frac{LA}{LN-INT}$$

where LA is the average length of hospital stay from admission to the day of the PPS of all patients, LN is the average length of hospital stay from admission to the day of the PPS of patients who acquired at least one considered HAI, and INT is the average number of days between admission and onset of the first HAI for patients who acquired one of the considered HAIs. Estimates of LN − INT, which reflect the length of infection from date of onset to day of PPS (LOI), were obtained for each considered HAI category.

We obtained LA from hospital-level data (number of patient-days divided by number of discharges for the year preceding the PPS in participating hospitals), and LA resulted in 7.87 days (interquartile range (IQR): 6.63–8.68).

The LOI was estimated for each patient as the difference between date of PPS and date of HAI onset + 1 (obtained from patient-level data). We expressed average LOI as the average of mean LOI and median LOI. According to analyses performed by the ECDC, this method offered the best mathematical relationship between length of stay up to the day of PPS (obtained from patient-level data) and LA (derived from hospital-level data, as previously described) [11]. Average LOI estimates in our sample ranged from 4.9 days for HA-UTI to 8.02 days for SSI.

#### Step 3: Incidence – projection to the Italian hospitalised population

We obtained the total number of acute-care hospital discharges in Italy in 2022 from the Ministry of Health’s annual report [14]. We projected the age, sex and McCabe score distribution of the patients in the PPS sample to the total number of hospital discharges. Stratum-specific incidence estimates were then applied to obtain the annual number of patients developing an HAI, for each considered HAI category.

#### Step 4: Burden of disease in disability-adjusted life years

We used the 2.0.0 version of the BCoDE Toolkit to build disease models for each considered HAI [15]. We customised the HA-UTI model based on results of a previous systematic review, also employed by Cassini et al. [1,16]. Inputs for model parameters included uncertainty intervals (UIs), and were incorporated as uniform (two variables) or PERT distribution (three variables) where applicable.

We used data from the Italian National Institute of Statistics (ISTAT) on Italian life expectancy in 2022 to populate the models [17]. Following the methodology described by Cassini et al., we built three models for each HAI type; one model was built for each McCabe score stratum setting median life expectancy at 0.5 years, 3 years and regular life expectancy for McCabe scores 3, 2 and 1 respectively. Stratum-specific incidence data obtained from the previous steps, referring to the national hospitalised population, including 95% UIs, were incorporated in models as PERT distributions [1]. Inputted data are available as Supplement 1. Models were run at 10,000 iterations of the Monte Carlo simulations, applying a 3.5% annual discounting rate [18]. For each output, point estimates and 95% UIs based on input uncertainties were calculated.

To obtain general population estimates, the annual number of cases, deaths and DALYs were normalised by the total Italian population [17]. We chose this approach to avoid making assumptions on the McCabe score distribution in the general population.

### Outcome measures

We considered the following outcome measures:

- annual number of HAIs in 2022, incidence per 100,000 hospitalised patients, incidence per 100,000 general population (calculated as the annual number of HAIs normalised by the Italian population, assuming only hospitalised patients can acquire an HAI as measured in the PPS),
- annual number of attributable deaths in 2022, mortality per 100,000 hospitalised patients, mortality per 100,000 general population,
- DALYs per case (composite measure of years lived with disability (YLDs), and years of life lost (YLLs)), number and rate per 100,000 hospitalised patients and general population of DALYs, YLLs and YLDs [19].

## Results

The Italian representative sample included 19,735 patients, of whom 18,397 had complete data and were included in the analyses. Table 1 reports clinical and demographic characteristics used to stratify patients in the models. Of the five considered HAIs, the most prevalent was HA-BSI, however, the weighted prevalence of HA-UTI was the highest (Table 2).

**Table 1:** Demographic and clinical characteristics of patients included in the representative sample, Italy, 2022 (n = 18,397)^a^

Characteristic	n	%
Age (years)		
0–4	1,328	7.22
5–19	652	3.54
20–44	2,349	12.77
45–64	4,038	21.95
65–85	7,667	41.68
> 85	2,363	12.84
Sex		
Male	9,573	52.04
Female	8,824	47.96
McCabe score		
Non-fatal	13,395	72.81
Ultimately fatal	3,671	19.95
Rapidly fatal	1,331	7.24

^a^ 1,338 patients were excluded from analyses due to incomplete data regarding: age, sex, McCabe score, HAI status and length of stay (respectively 92, 4, 5, 6 and 1,240 patients).

**Table 2:** Crude and inverse probability weighted healthcare associated infections prevalence estimates calculated based on data from the representative sample, Italy, 2022 (n = 18,397)^a^

HAI prevalence	Crude, estimate (95% CI)	Weighted, estimate (95% CI)^b^
HA-BSI	1.69 (1.5–1.88)	0.53 (0.43–0.64)
HA-UTI	1.62 (1.44–1.81)	0.66 (0.55–0.79)
HAP	1.56 (1.39–1.75)	0.65 (0.54–0.78)
SSI	1.13 (0.98–1.29)	0.48 (0.39–0.59)
HA-CDI	0.47 (0.37–0.58)	0.17 (0.12–0.24)
**All**	**6.46 (6.11–6.83)**	**2.49 (2.27–2.73)**

CI: mid-P Clopper–Pearson confidence interval; HA-BSI: healthcare-associated bloodstream infections; HA-CDI: healthcare-associated *Clostridioides difficile* infections; HAP: healthcare-associated pneumonia; HA-UTI: healthcare-associated urinary tract infections; SSI: surgical site infections.

^a^ 1,338 patients were excluded from analyses due to incomplete data regarding: age, sex, McCabe score, HAI status and length of stay (respectively 92, 4, 5, 6 and 1,240 patients).

^b^ 14 further patients were excluded from analyses due to missing length of infection data.

Table 3 reports the annual burden of HAIs in 2022. Full output results stratified per HAI type and McCabe score are available in Supplement 2. Considering naïve estimates, the highest number of cases in 2022 was found for HA-UTI, followed by HAP, whereas the highest number of attributable deaths resulted for HA-BSI.

**Table 3:** Annual burden of five types of healthcare-associated infections, based on naïve and inverse probability-weighted calculations, Italy, 2022 (estimates based on the national representative sample, n = 18,397, and projected on the Italian population, n = 58,997,201)

HAI category	Number of cases, estimate (95% UI)		Number of attributable deaths, estimate (95% UI)		Number of YLLs, estimate (95% UI)		Number of YLDs, estimate (95% UI)		Number of DALYs, estimate (95% UI)	
	Crude	Weighted	Crude	Weighted	Crude	Weighted	Crude	Weighted	Crude	Weighted
HA-BSI	152,975.44 (141,305.76- 164,883.08)	70,953.27 (63,649.66–78,533.16)	20,938.61 (18,846.88–23,340.17)	9,656.95 (8,435.72–11,023.27)	299,892.13 (249,617.52–348,832.99)	143,636.97 (112,592.92–180,399.62)	58,463.46 (51,254.88–66,891.38)	28,219.19 (22,961.61–34,618.88)	358,855.83 (300,826.09–416,676.44)	171,771.45 (136,128.02–213,060.55)
HA-UTI	226,006.58 (208,951.06–244,886.89)	126,030.04 (113,784.47–139,079.17)	4,156.36 (3,572.33–4,793.76)	2,324.02 (1,962.28–2,726.74)	59,248.88 (48,425.34–71,986.87)	30,827.19 (24,766.6–38,508.49)	35,674.85 (29,452.16–43,429.83)	18,601.6 (14,895.94–22,914)	94,680.81 (78,119.58–114,889.6)	49,572.62 (39,451.75–61,305.47)
HAP	164,542,.59 (151,630.69–177,769.78)	94,471.82 (84,672.43–104,001.79)	5,925.27 (5,444.05–6,421.91)	3,396.57 (3,036.79–3,772.01)	50,506.33 (44,736.95–56,769.24)	45,897.96 (37,785.4–53,707.71)	26,908.31 (23,801.39–30,161.58)	23,927.21 (19,817.45–28,353.72)	77,407.31 (68,764.21–86,594.18)	69,818.5 (57,311.82–82,693.47)
SSI	103,347.82 (94,702.3–113,374.54)	59,654.24 (52,458.53–66,415.15)	2,355.77 (2,124.61–2,621.86)	1,305.33 (1,115.04–1,495.56)	30,854.71 (27,599.77–34,599.08)	16,741.65 (14,091.38–19,461.94)	222.38 (184.6–262.16)	128.15 (103.91–153.49)	31,084.67 (27,738.81–34,791.95)	16,881.13 (14,200.01–19,607.89)
HA-CDI	80,477.98 (71,281.06–90,053.23)	53,267.62 (45,961.4–60,856.8)	4,413.87 (3,534.14–5,390.19)	2,921.33 (2,335.15–3,597.98)	36,656.27 (27,415.14–47,447.19)	24,008.42 (17,382.7–31,672.78)	1,786.66 (1,370.03–2,254.14)	1,166.28 (884.13–1,502.85)	38,448.95 (29,069.97–49,464.5)	25,175.06 (18,417.19–32,999.66)
**All**	**727,350.41 (667,870.88–790,967.52)**	**404,377 (360,526.48–448,886.06)**	**37,789.89 (33,552.01–42,567.89)**	**19,604.19 (16,884.99–22,615.54)**	**477,158.31 (397,794.71–559,635.37)**	**261,112.19 (206,619–323,750.54)**	**123,055.56 (106,063.05–142,999.58)**	**72,042.42 (58,663.05–87,542.94)**	**600,477.58 (504,518.66–702,416.68)**	**333,218.77 (265,508.79–409,667.05)**

DALYs: disability-adjusted life years; HA-BSI: healthcare-associated bloodstream infections; HA-CDI: healthcare-associated *Clostridioides difficile* infections; HAP: healthcare-associated pneumonia; HA-UTI: healthcare-associated urinary tract infections; SSI: surgical site infections; UI: uncertainty interval; YLDs: years lived with disability; YLLs: years of life lost.

**Figure 2:**
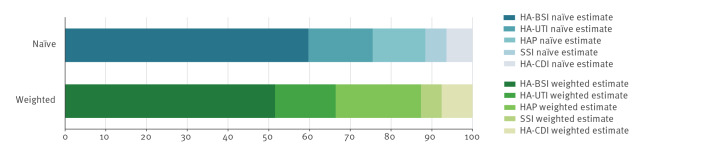
Percentage of annual burden in disability-adjusted life years attributable to five types of healthcare-associated infections, based on naïve and inverse probability weighted calculations, Italy, 2022 (estimates based on the national representative sample, n = 18,397, and projected on the Italian population, n = 58,997,201)

HA-BSI: healthcare-associated bloodstream infections; HA-CDI: healthcare-associated *Clostridioides difficile* infections; HAP: healthcare-associated pneumonia; HA-UTI: healthcare-associated urinary tract infections; SSI: surgical site infections.

DALYs per case were lowest for SSI and HA-UTI: 0.3 (95% UI: 0.28–0.32) and 0.42 (95% UI 0.35–0.50), respectively, and were highest for HA-BSI: 2.35 (95% UI: 2.01–2.69). As depicted in Figure 2, HA-BSI accounted for the majority of total DALYs (59.76%). The highest amount of DALYs within the HA-BSI category was attributable to cases among patients in the non-fatal McCabe score stratum (326,121.54 DALYs; 95% UI: 269,608.91–386,017.04). In terms of HA-BSI cases in 2022, 43.30% occurred among patients in the non-fatal McCabe score stratum. Considering all HAIs, 79.46% of DALYs were attributable to YLLs. This proportion ranged from 62.58% for HA-UTI to 99.26% for SSI.

Considering weighted estimates, the highest number of cases in 2022 was found for HA-UTI and HAP, and the highest number of attributable deaths for HA-BSI. The HA-BSI accounted for a smaller percentage of DALYs (51.55%) compared with calculations based on crude prevalence, whereas HAP increased in percentage of DALYs (20.95% vs 12.89%, Figure 2). In both estimations, HA-UTI, SSI and HA-CDI DALY percentages remained relatively stable.

**Figure 3:**
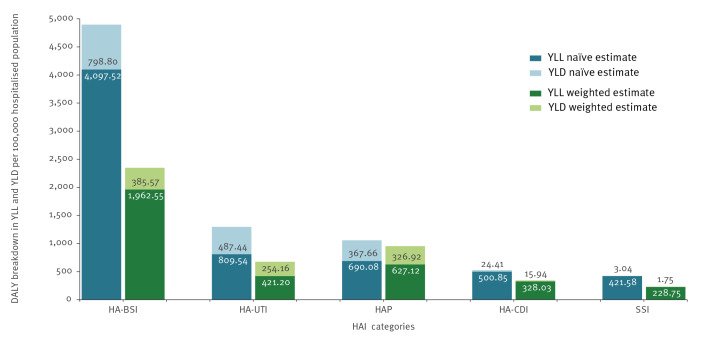
Annual burden in disability-adjusted life years per 100,000 hospitalised population of five types of healthcare-associated infection, as sum of years lived with disability and years of life lost, based on naïve and inverse probability weighted calculations, Italy, 2022 (estimates based on the national representative sample, n = 18,397, and projected on the Italian population, n = 58,997,201)

DALYs: disability-adjusted life years; HA-BSI: healthcare-associated bloodstream infections; HA-CDI: healthcare-associated *Clostridioides difficile* infections; HAP: healthcare-associated pneumonia; HA-UTI: healthcare-associated urinary tract infections; SSI: surgical site infections; YLDs: years lived with disability; YLLs: years of life lost.

Table 4 reports the annual burden of HAIs expressed per 100,000 hospitalised and general population, and Figure 3 shows DALYs broken down in YLLs and YLDs per 100,000 hospitalised population. When comparing results of Table 4 and Figure 3, it must be considered that the sum of YLDs and YLLs per 100,000 hospitalised population (shown in Figure 3) slightly differ from the total number of DALYs per 100,000 hospitalised population (Table 4), as the sum of the point estimates of two distributions (resulting from separate simulations for YLDs and YLLs) is not necessarily equal to the point estimate of the sum of the distributions (DALYs calculated as the sum of YLDs and YLLs at each simulation) [20]. In both estimations, HA-BSI had the highest burden in terms of DALYs per 100,000 hospitalised and general population. As depicted in Figure 3, HA-BSI DALY estimates based on crude and weighted prevalence showed the biggest difference, whereas the two estimates for HAP were similar. In both calculations, HA-UTI resulted in the highest incidence per 100,000 hospitalised and general population, followed by HAP and HA-BSI, and HA-BSI had the highest attributable mortality.

**Table 4:** Annual burden per 100,000 population of five types of healthcare-associated infections, based on naïve and inverse probability weighted calculations, Italy, 2022 (estimates based on the national representative sample, n = 18,397, and projected on the Italian population, n = 58,997,201)

HAI category	DALYs per 100,000 hospitalised population, estimate (95% UI)		DALYs per 100,000 general population, estimate (95% UI)		Incidence per 100,000 hospitalised population, estimate (95% UI)		Incidence per 100,000 general population, estimate (95% UI)		Mortality per 100,000 hospitalised population, estimate (95% UI)		Mortality per 100,000 general population, estimate (95% UI)	
	Crude	Weighted	Crude	Weighted	Crude	Weighted	Crude	Weighted	Crude	Weighted	Crude	Weighted
HA-BSI	4,903.16 (4,110.28–5,693.18)	2,346.96 (1,859.96–2,911.11)	608.26 (509.9–706.26)	291.15 (230.74–361.14)	2,090.15 (1,930.7–2,252.85)	969.46 (869.66–1,073.02)	259.29 (239.51–279.48)	120.27 (107.89–133.11)	286.09 (257.51–318.9)	131.95 (115.26–150.61)	35.49 (31.95–39.56)	16.37 (14.3–18.68)
HA-UTI	1,293.65 (1,067.37–1,569.77)	677.33 (539.04–837.64)	160.48 (132.41–194.74)	84.03 (66.87–103.91)	3,088.00 (2,854.96–3,345.96)	1,721.99 (1,554.67–1,900.28)	383.08 (354.17–415.08)	213.62 (192.86–235.74)	56.79 (48.81–65.5)	31.75 (26.81–37.26)	7.05 (6.06–8.13)	3.94 (3.33–4.62)
HAP	1,057.64 (939.55–1,183.16)	953.95 (783.07–1,129.87)	131.21 (116.56–146.78)	118.34 (97.14–140.17)	2,248.19 (2,071.78–2,428.92)	1,290.8 (1,156.9–1,421.01)	278.90 (257.01–301.32)	160.13 (143.52–176.28)	80.96 (74.38–87.74)	46.41 (41.49–51.54)	10.04 (9.23–10.89)	5.76 (5.15–6.39)
SSI	424.72 (379–475.37)	230.65 (194.02–267.91)	52.69 (47.02–58.97)	28.61 (24.07–33.24)	1,412.07 (1,293.95–1,549.07)	815.07 (716.76–907.45)	175.17 (160.52–192.17)	101.11 (88.92–112.57)	32.19 (29.03–35.82)	17.84 (15.24–20.43)	3.99 (3.6–4.44)	2.21 (1.89–2.53)
HA-CDI	525.34 (397.19–675.85)	343.97 (251.64–450.88)	65.17 (49.27–83.84)	42.67 (31.22–55.93)	1,099.6 (973.93–1,230.42)	727.81 (627.98–831.5)	136.41 (120.82–152.64)	90.29 (77.9–103.15)	60.31 (48.29–73.65)	39.91 (31.91–49.16)	7.48 (5.99–9.14)	4.95 (3.96–6.1)
**All**	**8,204.51 (6,893.39–9,597.33)**	**4,552.87 (3,627.73–5,597.4)**	**1,017.81 (855.16–1,190.59)**	**564.8 (450.04–694.38)**	**9,938.01 (9,125.32–10,807.23)**	**5,525.12 (4,921.98–6,133.27)**	**1,232.86 (1,132.04–1,340.69)**	**685.42 (611.09–760.86)**	**516.33 (458.02–581.62)**	**267.86 (230.7–309)**	**64.05 (56.82–72.15)**	**33.23 (28.62–38.33)**

DALYs: disability-adjusted life years; HA-BSI: healthcare-associated bloodstream infections; HA-CDI: healthcare-associated *Clostridioides difficile* infections; HAP: healthcare-associated pneumonia; HA-UTI: healthcare-associated urinary tract infections; SSI: surgical site infections; UI: uncertainty interval; YLDs: years lived with disability; YLLs: years of life lost.

## Discussion

This study reports updated burden estimations for five major HAIs in Italy. The DALY estimations allow us to consider deaths and disabilities combined, providing a comprehensive assessment of HAI burden. With this metric, it is possible to compare national and international estimates for HAI burden over time and to make comparisons with other diseases and health conditions [4,19].

According to our results (weighted estimates), more than 400,000 cases of the five considered HAIs occur in Italy each year, causing almost 20,000 deaths per year. For comparison, the estimated number of influenza cases during the entire 2022/23 epidemic season in Italy was almost 14 million [21]. Notified COVID-19 cases and deaths in Italy in 2022 were around 20 million and 50,000, respectively [22].

Considering the five HAIs included in our study (weighted estimates), we calculated overall 564.8 DALYs per 100,000 general population, with an annual incidence of 685.42 cases and 33.23 deaths per 100,000 general population per year. The DALY estimations in this study are higher than previous projections of the burden of non-HA communicable diseases in Europe. According to findings of the BCoDE study from 2009 to 2013, an annual burden of 260 DALYs per 100,000 population was estimated in Europe for 31 infectious diseases listed in Decision 2119/98/EC with amendments, including 71.2 DALYs per 100,000 for influenza and 53.5 DALYs per 100,000 for tuberculosis, with an overall number of attributable deaths of 9.67 per 100,000 population per year [23]. The same study estimated an annual incidence for all 31 included diseases of 7,577 cases per 100,000 population, of which 5,887 were attributable to influenza [23]. In 2021, the Global Burden of Disease (GBD) study estimated the age-standardised DALY rate per 100,000 persons of all communicable, maternal, neonatal and nutritional diseases for high-income countries at 2,439.5, including 1,220.2 DALYs per 100,000 due to COVID-19 [24]. According to the GBD study, COVID-19 ranked second among the leading causes of DALYs in high-income countries of western Europe (Andorra, Austria, Belgium, Cyprus, Denmark, Finland, France, Germany, Greece, Iceland, Ireland, Israel, Italy, Luxembourg, Malta, Monaco, Netherlands, Norway, Portugal, San Marino, Spain, Sweden, Switzerland, United Kingdom).

Comparisons between these studies have limited value, due to the differences in methods, approaches and considered time periods. In particular, severe acute respiratory syndrome coronavirus 2 (SARS-CoV-2) in 2021 was an emerging pathogen, which caused an unprecedented initial impact on health systems across Europe [25]. Excluding COVID-19 due to the particular situation in 2021 and 2022, it would appear that even though HAIs only occur among a small subset of the population, and even if correcting life expectancy based on underlying health conditions, HAIs in Italy have a high burden on population health in comparison with other non-HA communicable diseases, with implications on the use of healthcare resources [1].

The HAI burden results in our study were higher than previous estimations for the European Union/European Economic Area (EU/EEA) which were around 261 DALYs and 512 cases per 100,000 population according to Cassini et al. (with a 3.5% annual time discount rate as applied in this study, and including HA neonatal sepsis) [1], and around 290 DALYs, 470 cases and 15 attributable deaths per 100,000 population according to Zacher et al., who employed an updated methodology [20]. In interpreting these results, in addition to methodological differences, it must be noted that the estimates by Cassini et al. and Zacher et al. were based on data from a previous PPS edition [1,20].

According to the 2022/23 ECDC PPS report, the prevalence of patients with at least one HAI in the EU/EEA did not increase much compared versus the 2016/17 and 2011/12 editions, remaining in the range of 5.8–6.2% (after accounting for differences in the subsequent protocols) [11,26]. In the latest edition, Italy ranked fifth among European countries in terms of overall HAI prevalence, which was estimated at 9.8%. Italian patients had the longest lengths of stay among participant EU/EEA countries, higher exposures to invasive devices and surgical procedures, and rapidly/ultimately fatal Mc Cabe scores were assigned more frequently than the EU/EEA average [11]. These findings are in line with our previous observation of a trend towards a broadening of indications for invasive procedures and an increase in the intensity of care in our country (data not shown), which could have repercussions on HAI risk.

Further, it must be considered that the use of broad-spectrum antimicrobial agents in Italy remains among the most extensive in Europe, and Italian AMR rates for most critical pathogens are generally much higher than EU/EEA averages [11]. Concerning the latter, a modelling analysis of the burden of infections caused by AMR bacteria of public health concern in 2015 found that Italy and Greece had the greatest disease burden in the EU/EEA, accounting for a combined 21.3% of total EU/EEA DALYs per 100,000 population [27].

The second objective of this study was to describe our adapted HAI burden estimation methodology, obtained from inverse probability-weighted calculations. The PPSs are limited by the fact that patients are not sampled from the population with equal probability: patients with longer at-risk times (i.e. lengths of stay) are more likely to be included than patients with shorter at-risk times. Further, HAI risk factors are generally not equally distributed among these two groups: patients with more risk factors tend to have longer lengths of stay. Therefore, the probability of being sampled in PPS is proportional to a patient’s time-at-risk. This leads to a selection bias known as ‘length bias’, which does not directly affect prevalence but affects the incidence and risk estimates obtained from naïve prevalence to incidence conversion. By weighting patients inversely proportional to their time-at-risk, patients with low and high inclusion probability are respectively up- and down-weighted in the reconstructed sample [8]. It must be noted that by adjusting inputs, the BCoDE toolkit can be used to obtain weighted burden estimates [15].

Results of naïve calculations in this study were higher but reasonably in line with our previous estimations based on 2017 data, with almost overlapping UIs [6]. Compared with naïve estimates, current weighted calculations reduced HAI burden by around half, in terms of incidence, attributable mortality and DALYs. The overestimation due to length biased sampling could in part explain the high burden found in our previous analysis, although results cannot be compared directly due to other updates in the applied methodology. In this study, the difference between naïve and weighted estimates was not equal among all considered HAIs, suggesting a different impact of length bias based on HAI type and respective LOI.

HA-BSI was the most frequently recorded HAI in the 2022 Italian PPS, accounting for 16.05% of all HAIs. In comparison, HA-BSI was the fourth most frequent HAI recorded at EU/EEA level (11.9% of HAIs). A possible explanation for this difference could be the aforementioned high exposure of Italian patients to invasive devices. The relative frequency of catheter-related infections among all HAIs measured in Italy was also among the highest in Europe [11]. After applying inverse probability weighting, HA-BSI ranked third in terms of prevalence after HA-UTI and HAP, which could indicate that selection bias disproportionally affects the HA-BSI category compared with other HAIs. In fact, in the Italian sample, patients with an HA-BSI on the day of the PPS had higher lengths of stay up to the day of the PPS compared with other HAI patients: a median of 22 days (IQR: 13–38) vs 16 days (IQR: 9–30) and 12.5 days (IQR: 8–25) for patients with HAP and HA-UTI, respectively (data not shown). Notwithstanding the correction provided by inverse probability weighting, even though the annual number of cases was higher for HA-UTI and HAP, HA-BSI had the highest number of attributable deaths and of DALYs per case, resulting in an overall higher burden. In our study, HA-BSI accounted for 63.85% of all DALYs, almost double the proportion estimated by Cassini et al. (28.9%) [1].

Several limitations of this study should be considered when interpreting results. As any modelling analysis, our study was limited by the representativeness of inputted data and the validity of our assumptions. Concerning the former, our sampling method ensured a proportional representation of Italian regions, however, participation within regions was voluntary. Further, in the weighted analyses, 14 patients were excluded, which could have led to a small underestimation of HAI burden in comparison to results of naïve calculations.

Concerning the latter, our calculations assumed that HAIs occur only among patients hospitalised in acute-care facilities, as this was the focus of the PPS. Our burden estimates did not include HAIs occurring in long-term care facilities or other non-acute settings and are therefore an underestimation of the overall HAI burden in Italy. Further, we only considered the same five major types of HAIs included in previous estimations. In particular, we did not include healthcare-acquired COVID-19, due to reservations about the applicability of case definitions, as previously discussed [12]. Finally, we did not account for patients with multiple HAIs (only the first HAI for each patient was considered). As HAIs were considered independently, our method does not account for changes in HAI risk based on having acquired previous HAIs.

The BCoDE methodology applies an incidence-based approach to estimate the present and future HAI burden over patients’ lifetime, which requires entering yearly HAI incidence in disease models. However, inputted data are derived from PPSs, which are often the most comprehensive available data source but are designed to measure disease prevalence. Therefore, a critical step of the workflow is prevalence to incidence conversion. In our study, we expressed average LOI as the average between mean LOI and median LOI, applying the methodology proposed by ECDC [11]. Cassini et al. previously employed a median estimator for LOI, and more recently mean and Grenander estimators have been proposed [1,20,28]. As found in analyses of European PPS3 data [11], the method we applied showed a good approximation of overall length of stay for all patients when obtained from hospital-level data (7.87 days; IQR: 6.63–8.68) vs that resulting from patient-level information (8.67 days).

It should be noted that the method we propose applies a double correction for length of stay: firstly, in the inverse probability weighting and secondly, through the Rhame and Sudderth formula, which also applies a correction for length of stay (through LOI). We cannot exclude a risk of over-adjustment through this method. However, we previously found the weighted method to be extremely accurate in estimating incidence rates from prevalence measurements for device-associated HAIs in ICUs [9]. Further research should be dedicated to comparing estimates obtained through the weighted method against those obtained with the Grenander estimates.

The Rhame and Sudderth formula has well known limitations that still apply to this study [13]. Its reliability has been questioned for SSIs in particular [29,30]. Given the high proportion of SSIs developing post discharge (up to 55% for some procedure categories), SSIs measured through PPSs may not accurately reflect the true occurrence of SSIs [31]. In addition, as post-discharge SSIs do not influence the length of initial hospitalisation, LOI measurement through LN – INT could be unreliable [29]. Finally, outcome trees employed in this study did not account for procedure-specific health outcomes and disability [32]. These factors could have led to a further underestimation of SSI burden in particular.

## Conclusion

This study provides updated burden estimates for five major HAIs in Italy, obtained through an evidence-based and transparent approach and based on the most recent data available from a nation-wide, comprehensive sample of hospitals. This study describes step-by-step an updated methodology for incorporating inverse probability weighting in burden estimations, which could lead to more valid results than those based on naïve calculations. This methodology could be routinely applied given its relative simplicity. Even after correcting for length-biased sampling, our study confirms the substantial burden of HAIs in Italy and renews the need to prioritise resources for infection prevention and control interventions.

## Data Availability

All data collected within the CCM project “Sostegno alla Sorveglianza delle infezioni correlate all’assistenza anche a supporto del PNCAR”, including those reported in this study, are owned by the Italian Ministry of Health. Datasets will be made available by the corresponding author upon reasonable request.

## Supplementary Data

114000 Supplement_1

## Supplementary Data

56000 Supplement_2
